# Supplementary figures and images for: Glycolytic flux sustains human Th1 identity and effector function via STAT1 glycosylation
Source: Life Sci Alliance. 2025 Nov 3;9(1):e202503315. doi: 10.26508/lsa.202503315 (PMC12583888; doi:10.26508/lsa.202503315)

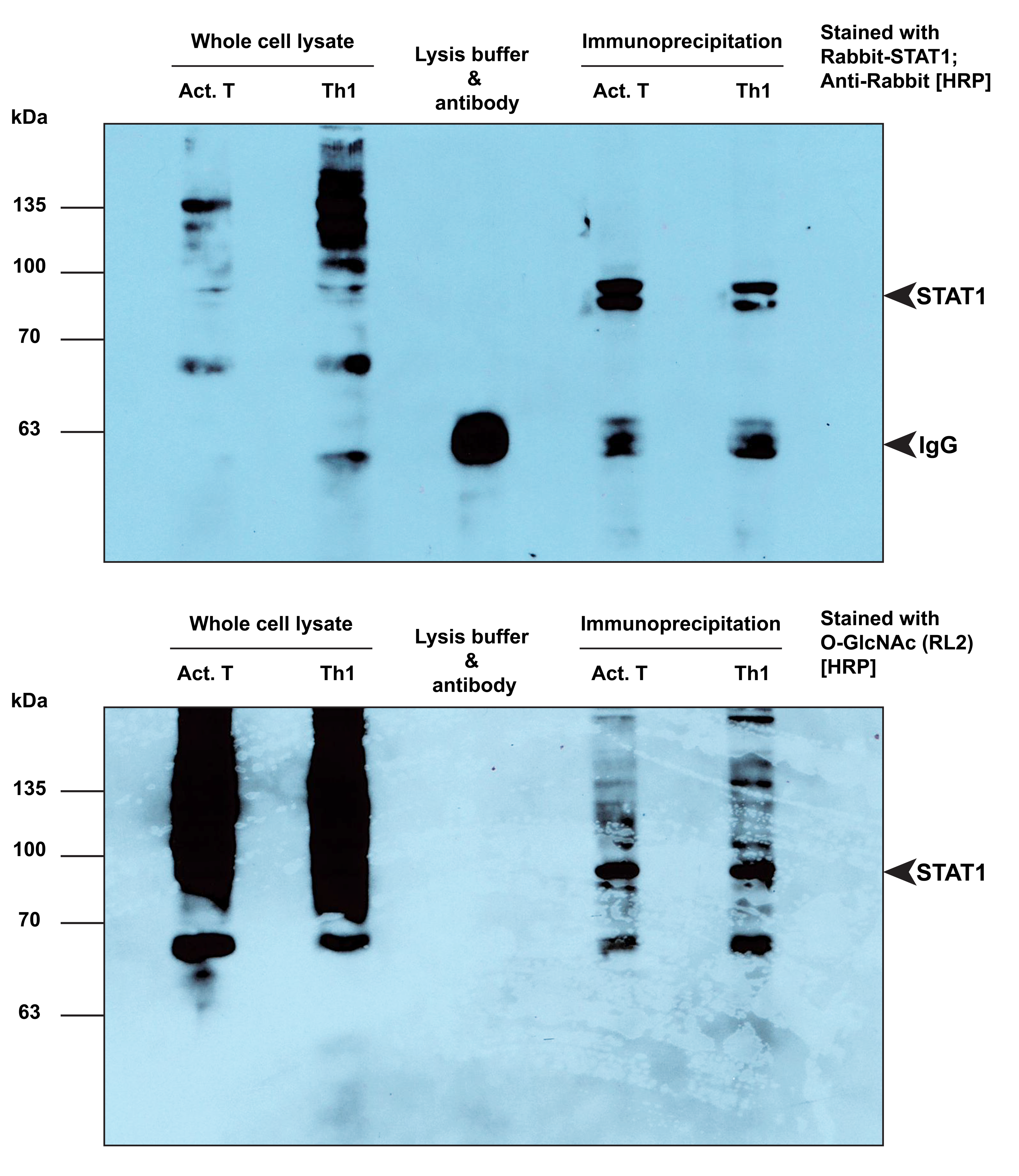

Supplement: Supplementary file 1 [file LSA-2025-03315_SdataF6.1.tif]

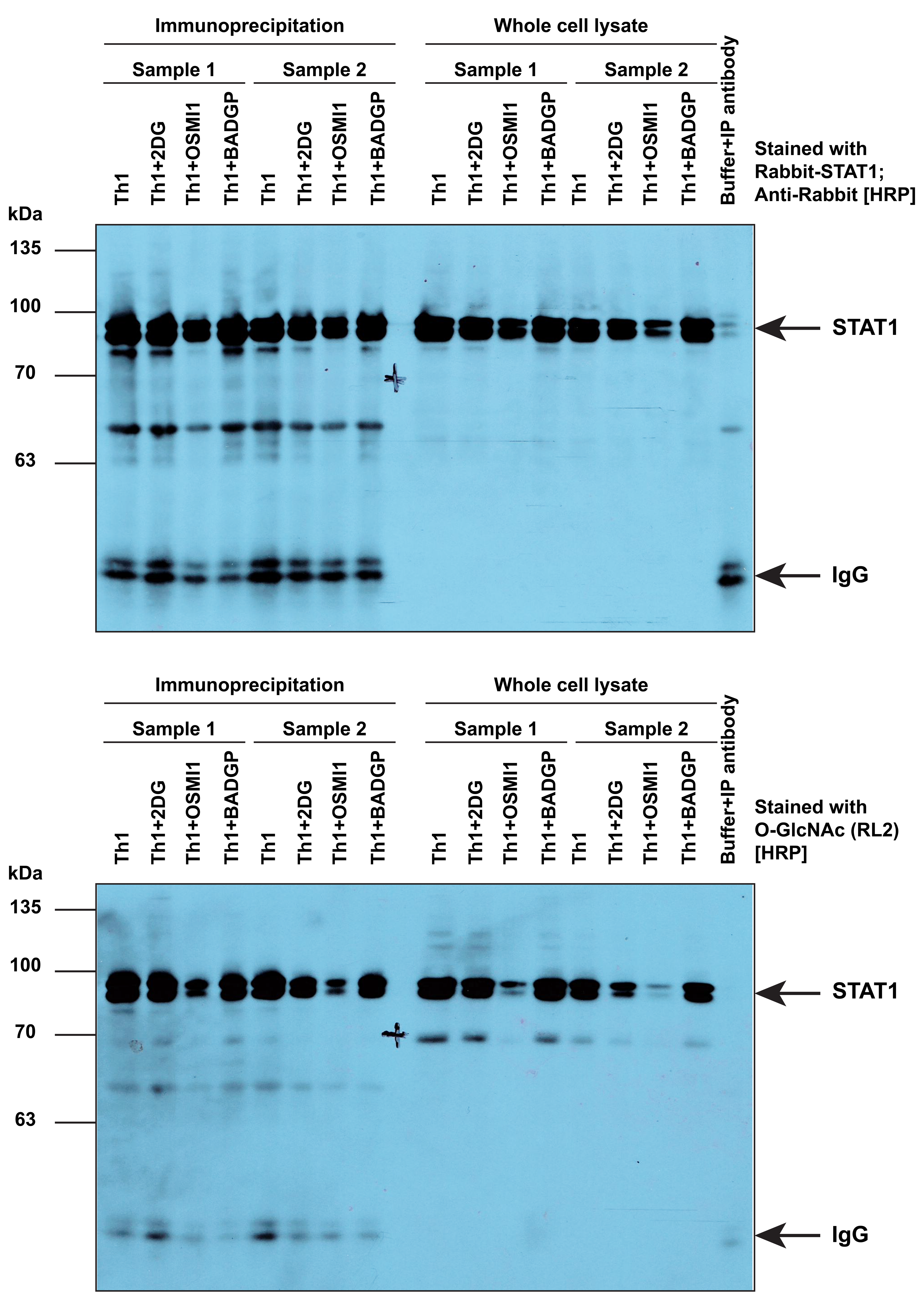

Supplement: Supplementary file 2 [file LSA-2025-03315_SdataF6.2.tif]
