# Supplementary material for: Glycolytic flux sustains human Th1 identity and effector function via STAT1 glycosylation
Source: Life Sci Alliance. 2025 Nov 3;9(1):e202503315. doi: 10.26508/lsa.202503315 (PMC12583888; doi:10.26508/lsa.202503315)
Supplement: Supplementary file 3 [file LSA-2025-03315_TableS1.docx]

**Table S1: Sequences of the homology directed repair templates**

| **Name** | **Sequences** |
| --- | --- |
| **LHA** | AGAGGTGAAACAGGAAGCGAGTGTCATTTTGTTTGGCTTCAGTTGG  AAGCGTGTTAAGAGACTCGAATTCTTTGCTGCTGTGTGCTGCTGTGT  GTGCACGGGTGTGTCTTCAAATGACCCCAAAGATGCCATGTATTTAG  ATTTTGGAGTTACAAGTAAATTTTAGTGAGTAAACAAGTTCACAAAT  GTGCATCTGTAAATAATGAAAATTGACTGTATTTCTCTTCCCCTACTGT  GAAAGCACCTGTGTGTCATATAAACTAGAATTGAACTTTGGGATGGA  CATATGTTTTAGTGCCACACTTGTGACTGGTGTCTCTGTAGTAACCCT  TAGATTTTGGGTGTTTTCTCTCTAGAATCTGTCCTTCTTCCTGACTCCA  CCATGTGCA |
| **STAT1 WT insert** | AGGTGGGCTCAGCTGTCAGAAGTGCTGTCCTGGCAGTTCTCTAGCG  TCACCAAAAGAGGGCTCAATGTTGACCAACTGAACATGTTGGGCGA  GAAGCTTTTAGGA |
| **STAT1 edited insert** | AGGTGGGCTCAGCTGGCCGAAGTGCTGTCCTGGCAGTTCTCTAGCG  TCGCAAAAAGAGGGCTCAATGTTGACCAACTGAACATGTTGGGCGA  GAAGCTTTTAGGA |
| **P2A** | GGCAGCGGCGCCACCAACTTCAGCCTGCTGAAGCAGGCCGGCGAC  GTGGAAGAGAACCCCGGGCCC |
| **EGFP** | ATGGTGAGCAAGGGCGAGGAGCTGTTCACCGGGGTGGTGCCCATC  CTGGTCGAGCTGGACGGCGACGTAAACGGCCACAAGTTCAGCGTG  TCCGGCGAGGGCGAGGGCGATGCCACCTACGGCAAGCTGACCCTG  AAGTTCATCTGCACCACCGGCAAGCTGCCCGTGCCCTGGCCCACCC  TCGTGACCACCCTGACCTACGGCGTGCAGTGCTTCAGCCGCTACCCC  GACCACATGAAGCAGCACGACTTCTTCAAGTCCGCCATGCCCGAAGG  CTACGTCCAGGAGCGCACCATCTTCTTCAAGGACGACGGCAACTACA  AGACCCGCGCCGAGGTGAAGTTCGAGGGCGACACCCTGGTGAACCG  CATCGAGCTGAAGGGCATCGACTTCAAGGAGGACGGCAACATCCTGG  GGCACAAGCTGGAGTACAACTACAACAGCCACAACGTCTATATCATGG  CCGACAAGCAGAAGAACGGCATCAAGGTGAACTTCAAGATCCGCCAC  AACATCGAGGACGGCAGCGTGCAGCTCGCCGACCACTACCAGCAGAA  CACCCCCATCGGCGACGGCCCCGTGCTGCTGCCCGACAACCACTACCT  GAGCACCCAGTCCGCCCTGAGCAAAGACCCCAACGAGAAGCGCGATC  ACATGGTCCTGCTGGAGTTCGTGACCGCCGCCGGGATCACTCTCGGCA  TGGACGAGCTGTACAAGTAA |
| **RHA** | CGATGGGCTCAGCTTTCAGAAGTGCTGAGTTGGCAGTTTTCTTCTGTCA  CCAAAAGAGGTCTCAATGTGGACCAGCTGAACATGTTGGGAGAGAAGC  TTCTTGGTAGAGAAGCTTCTTGGTATATGCATATTAACTTGTTATGTTTATA  AAAATTGAAATTCATAAAAATATCTCTCTAATTGCTCTTTTCCCCTCTGCTA  TTTTGTTAAAGGTAAAAAAGTACTAAAATCTGTCAGCTTTTCAAGCTATAG  TTTATTATAGCTAAGTGAGAATCATATGTCACCTTAGAAAGAAATATAGACC  TGATAACATTTAAATGAATCCGTTCCTCATTGTCTCATATTAAGATTTCTGA  GATGAATTCCCAAGGGAAAG |
