# Supplementary material for: Glycolytic flux sustains human Th1 identity and effector function via STAT1 glycosylation
Source: Life Sci Alliance. 2025 Nov 3;9(1):e202503315. doi: 10.26508/lsa.202503315 (PMC12583888; doi:10.26508/lsa.202503315)
Supplement: Supplementary file 4 [file LSA-2025-03315_TableS2.docx]

**Table S2: Raw data table of IFNγ MFI and** **percentage of Figure 7**

|  | **MFI** |  | **Percentage** |  |
| --- | --- | --- | --- | --- |
|  | **GFP^-^** | **GFP^+^** | **GFP^-^** | **GFP^+^** |
|  | 49107.89 | 49003.79 | 22.13 | 21.77 |
|  | 47878.46 | 45702.72 | 22.71 | 21.69 |
|  | 45916.34 | 44413.34 | 23.9 | 20.7 |
|  | 58961.07 | 56049.77 | 21.38 | 20.05 |
| **STAT1^mut^** | 60217.61 | 57318.04 | 21.25 | 19.75 |
|  | 52302.02 | 48913.83 | 22.17 | 20 |
|  | 228859.3 | 224917.4 | 54.64 | 51.76 |
|  | 230499.1 | 232519.5 | 49.23 | 39.45 |
|  | 265119.2 | 246865.8 | 55.34 | 47.96 |
|  | 270109.6 | 215533.5 | 48.33 | 51.34 |
|  |  |  |  |  |
|  | 50724.82 | 50386.55 | 21.71 | 19.54 |
|  | 41904.89 | 42725.93 | 21.17 | 20.56 |
|  | 43506.4 | 43880.14 | 25.71 | 20.64 |
|  | 223427.5 | 212567.31 | 19.69 | 18.85 |
| **STAT1^wt^** | 218653.9 | 192745.52 | 7.96 | 5.72 |
|  | 212302.8 | 242389.6 | 22.57 | 22.17 |
|  | 198256.3 | 215767.9 | 55.33 | 48.71 |
|  | 59220.62 | 55049.9 | 52.98 | 47.28 |
|  | 44088.56 | 34217.34 | 55.78 | 51.04 |
