# Supplementary material for: Glycolytic flux sustains human Th1 identity and effector function via STAT1 glycosylation
Source: Life Sci Alliance. 2025 Nov 3;9(1):e202503315. doi: 10.26508/lsa.202503315 (PMC12583888; doi:10.26508/lsa.202503315)
Supplement: Supplementary file 5 [file LSA-2025-03315_TableS3.docx]

**Table S3: Reagents**

| **Products** | **Manufacturers** | **Catalogue No.** |
| --- | --- | --- |
| 2-Desoxy-D-Glucose (2DG). | Sigma - Aldrich | D8375 |
| 2-Mercaptoethanol | Gibco™, Thermo Fisher Scientific | 21985023 |
| 3PO | Sigma - Aldrich | SML1343-5MG |
| Agarose | PeqLab | 732-2789 |
| Albumin fraction V ( BSA ) | Carl Roth | 8076.4 |
| Albutein 50 g/l Infusionslösung | Grifols Deutschland GmbH | 10408446 |
| Antimycin A | Sigma - Aldrich | A - 8674 |
| APS | Carl Roth | 9592.3 |
| BD Cytofix™ Fixation Buffer | BD | 554655 |
| BD GolgiPlug™ | BD | 555029 |
| BD Phosflow™ Perm Buffer III | BD | 558050 |
| BSA, fatty acid-free | Sigma - Aldrich | A3803 |
| DAPI | Carl Roth | 28717-90-3 |
| DMSO | Molecular Probes | C10634 |
| Ethidium bromide | Carl Roth | 2218 |
| EtOH | Merck | 1.00983.2500 |
| FCCP | Sigma - Aldrich | C2920 |
| Fetal Bovine Serum | Gibco™, Thermo Fisher Scientific | 16000044 |
| Glucose | Carl Roth | HN06.3 |
| Glutaraldehyde | Carl Roth | 4995.1 |
| Glycerin | ROTIPURAN^®^, Carl Roth | 200-289-5 |
| Hydrochloric acid fuming 37% | Merck Millipore | 113386 |
| Ionomycin calcium salt | Sigma - Aldrich | I3909-1ML |
| L-Glutamine (200 mM) | Gibco™, Thermo Fisher Scientific | 25030081 |
| Milk Powder | Carl Roth | T145.3 |
| Mowiol 4-88 | Calbiochem | 47-590-4100GM |
| NaN_3_ | Carl Roth | 26628-22-8 |
| NP 24 - KLH | Biosearch Technologies | N - 5060-5 |
| Oligomycin | Sigma - Aldrich | 75351 |
| OSMI1 | Sigma - Aldrich | SML1621-5MG |
| PageRuler Prestained | Thermo Scientific | 26616 |
| p-Cumaric acid | Carl Roth | 9908.2 |
| Penicillin - streptomycin | Gibco | 15140-122 |
| Penicillin-Streptomycin (10,000 U/mL) | Gibco™, Thermo Fisher Scientific | 15140122 |
| Phosphate - Buffered Saline (PBS), 1x | Gibco™, Thermo Fisher Scientific | 14190-094 |
| PMSF | Sigma - Aldrich | 329-98-6 |
| Poly-D-Lysine | Sigma - Aldrich | P6407 |
| Ponceau S | Carl Roth | 5938.1 |
| Proteinase K | Peqlab | 04-1076 |
| Rotenone | Sigma - Aldrich | R8875 |
| RPMI1640 | Gibco™, Thermo Fisher Scientific | 11875093 |
| SDS ultra pure | Carl Roth | 205-788-1 |
| Seahorse XF RPMI medium, pH 7.4 | Agilent Technologies | 103576-100 |
| Sodium chloride | Carl Roth | 231-598-3 |
| Sodium iodoacetate | Sigma - Aldrich | I2512-25G |
| Sodium oxalate | Sigma - Aldrich | 379735-5G |
| Sodium hydroxide | Merck Millipore | 106462 |
| Sodium Pyruvate | Gibco™, Thermo Fisher Scientific | 11360070 |
| TEMED | Carl Roth | 2367.3 |
| Tris | Carl Roth | 4855.3 |
| Trizma Base | Sigma-Aldrich | T1503 |
| Tween - 20 | Carl Roth | P1379 |
