# Supplementary material for: Glycolytic flux sustains human Th1 identity and effector function via STAT1 glycosylation
Source: Life Sci Alliance. 2025 Nov 3;9(1):e202503315. doi: 10.26508/lsa.202503315 (PMC12583888; doi:10.26508/lsa.202503315)
Supplement: Supplementary file 6 [file LSA-2025-03315_TableS4.docx]

**Table S4: List of commercial kits**

| **Products** | **Manufacturers** | **Catalog No.** |
| --- | --- | --- |
| Naive CD4+ T Cell Isolation Kit II, human | Miltenyi | 130-094-131 |
| PyroMAT™ System | Merck Millipore | D5047 |
| Seahorse Extracellular Flux Analysis Kit | Agilent Technologies | 103793-100 |
| Seahorse XF Real-Time ATP Rate Assay Kit | Agilent Technologies | 103592-100 |
| VersaComp Antibody Capture Kit | Beckman Colter Life sci | B22804 |
