# Supplementary material for: Glycolytic flux sustains human Th1 identity and effector function via STAT1 glycosylation
Source: Life Sci Alliance. 2025 Nov 3;9(1):e202503315. doi: 10.26508/lsa.202503315 (PMC12583888; doi:10.26508/lsa.202503315)
Supplement: Supplementary file 7 [file LSA-2025-03315_TableS5.docx]

**Table S5: PCR**

| **Products** | **Manufacturers** | **Catalog No.** |
| --- | --- | --- |
| DNA/RNA dye, peqGREEN | VWR Peqlab | 732-3196 |
| dNTP Mix (10 mM each) | Thermo Fisher Scientific | R01922 |
| GeneRuler 1 kb DNA Ladder | Thermo Fisher Scientific | SM0311 |
| Q5 high-fidelity polymerase | New England Biolabs | M0491S |
| MinElute PCR Purification Kit | Qiagen | 28004 |
| RT-PCR Grade Water | Thermo Fisher Scientific | AM9935 |
