# Supplementary material for: Glycolytic flux sustains human Th1 identity and effector function via STAT1 glycosylation
Source: Life Sci Alliance. 2025 Nov 3;9(1):e202503315. doi: 10.26508/lsa.202503315 (PMC12583888; doi:10.26508/lsa.202503315)
Supplement: Supplementary file 8 [file LSA-2025-03315_TableS6.docx]

**Table S6: List of antibodies and cytokines**

| **Products** | **Manufacturers** | **Catalog No.** |
| --- | --- | --- |
| Alexa Fluor 647 Anti-O-Linked N-Acetylglucosamine antibody [RL2] (ab201994) | Abcam | ab201994 |
| Alexa Fluor 647 Mouse anti-Total Stat1 (N-Terminus) | BD Biosciences | 558560 |
| APC anti-human CD4 Antibody | BioLegend | 317416 |
| APC/Cyanine7 anti-human IFN-γ Antibody | Biolegend | 506524 |
| BD Pharmingen™ Human BD Fc Block™ | BD | 564219 |
| Brilliant Violet 421™ anti-human IL-4 Antibody | BioLegend | 500826 |
| FITC anti-human CD3 Antibody | BioLegend | 300406 |
| FITC anti-human CD45RA Antibody | BioLegend | 304148 |
| FITC anti-human IL-17A Antibody | BioLegend | 512304 |
| FITC OGT1 Polyclonal Antibody | Invitrogen | OGT1-FITC |
| FITC Phospho-Jak2 (Tyr1007, Tyr1008) Recombinant Rabbit Monoclonal Antibody (JAK2Y10071008-PB6), | Thermo Fisher Sci. | MA5-37198 |
| Human IL-12, premium grade | Miltenyi Biotec | 130-096-705 |
| Human IL-2 IS, research grade | Miltenyi Biotec | 130-097-743 |
| Human IL-4 Antibody | R&D System | MAB204-100 |
| Naive CD4+ T Cell Isolation Kit II, human | Miltenyi | 130-094-131 |
| O-GlcNAc Antibody (RL2) [HRP] | Novus Biologicals | NB300-524H |
| O-GlcNAc Antibody (RL2) [HRP] | Novus Biologicals | NB300-524H |
| O-GlcNAc Monoclonal Antibody (RL2), Alexa Fluor™ 488, eBioscience™ | Thermo Fisher | 53-9793-42 |
| PE anti-STAT1 Phospho (Ser727) Antibody | Biolegend | 686404 |
| PE Mouse Anti-Stat1 (pY701) | BD Biosciences | 612564 |
| PE Phospho-Jak1 (Tyr1022, Tyr1023) Recombinant Rabbit Monoclonal Antibody | Invitrogen | MA5-36891 |
| PerCP/Cyanine5.5 anti-T-bet Antibody | BioLegend | 644806 |
| Phospho-Stat1 (Ser727) Antibody #9177 | Cell Signaling Technology | 9177S |
| Phospho-Stat1 (Tyr701) (58D6) Rabbit mAb #9167 | Cell signaling | 9167S |
| Purified anti-human IL-4 Antibody | BioLegend | 500802 |
| Stat1 (9H2) Mouse mAb #9176 | Cell signaling | 9176S |
| Stat1 Antibody #9172 | Cell signaling | 9172S |
| STAT4 pY693 Antibody, anti-human, FITC, REAfinity™ | Miltenyi Biotec | 130-114-491 |
| Ultra-LEAF™ Purified anti-human CD28 Antibody | BioLegend | 302934 |
| Ultra-LEAF™ Purified anti-human CD3 Antibody | BioLegend | 300438 |
| Zombie Aqua™ Fixable Viability Kit | BioLegend | 423101 |
