# Supplementary material for: Glycolytic flux sustains human Th1 identity and effector function via STAT1 glycosylation
Source: Life Sci Alliance. 2025 Nov 3;9(1):e202503315. doi: 10.26508/lsa.202503315 (PMC12583888; doi:10.26508/lsa.202503315)
Supplement: Supplementary file 9 [file LSA-2025-03315_TableS7.docx]

**Table S7: Buffer solutions and medium**

| **Reagent** | **Composition** |
| --- | --- |
| Anode buffer | dH_2_O  25mM Tris  20ml/100ml MeOH |
| Antibody dilution | PBS  3g/100ml BSA  0.01ml/100ml Tween-20  0.1ml/100ml NaCl_3_ |
| Cathode buffer | dH_2_O  40mM 6-Aminocaproic acid  20ml/100ml MeOH |
| Cell sorting buffer (MACS) | PBS  2% Human Serum Albumin  5mM EDTA |
| ECL | 2 ml 1M Tris pH 8.5  17.7 ml H_2_O  90 µl 90 mM p-Cumaric acid  200 µl 200 mM Luminol  6 µl H_2_O_2_ |
| FACS buffer | PBS  2% FCS  5ml 100mM EDTA |
| Ponceau S | dH_2_O  1g/l Ponceau S |
| R10 Medium | RPMI1640  2mM L-Glutamine  100U/ml Penicillin  100U/ml Streptomycin  50µM 2-Mercaptoethanol |
| Seahorse ATP analysis medium | 1mM Sodium pyruvate  2mM L-Glutamine  10mM Glucose |
| Seahorse cell culture plate coating buffer | dH_2_O  100ng/ml Poly-D-lysine |
| Seahorse glyco-stress test medium | 1mM Sodium pyruvate  2mM L-Glutamine |
| Seahorse mito-stress test medium | 1mM Sodium pyruvate  2mM L-Glutamine  10mM Glucose |
| TBS-T | dH_2_O  40mM Tris/HCL pH 8.5  20mM Sodium acetate  1mM EDTA |
