# Supplementary material for: Glycolytic flux sustains human Th1 identity and effector function via STAT1 glycosylation
Source: Life Sci Alliance. 2025 Nov 3;9(1):e202503315. doi: 10.26508/lsa.202503315 (PMC12583888; doi:10.26508/lsa.202503315)
Supplement: Supplementary file 10 [file LSA-2025-03315_TableS8.docx]

**Table S8: List of software**

| **Name - Version** | **Utilization** |
| --- | --- |
| Adobe Illustrator 2017 | Illustrations |
| GraphPad Prism - 9.0.0 | Statistical analysis and data presentation |
| ImageJ - Fiji - 1.53t | Western Blot analysis |
| Kaluza Analysis - 2.1 | Flow Cytometry Analysis |
| Microsoft Excel 2019 | Data management |
| Microsoft Word 2019 | Manuscript writing and presentation |
| Wave 2.6.3.5 | Seahorse analysis |
| Zotero - 6.0.30 | Citation management |
